# Construction and Validation of Newly Adapted Sport-Specific Anaerobic Diving Tests

**DOI:** 10.3390/sports12040110

**Published:** 2024-04-18

**Authors:** Ivan Drviš, Dario Vrdoljak, Goran Dujić, Željko Dujić, Nikola Foretić

**Affiliations:** 1 Faculty of Kinesiology, University of Zagreb, 10000 Zagreb, Croatia; ivan.drvis@kif.unizg.hr; 2 Faculty of Kinesiology, University of Split, 21000 Split, Croatia; darvrd@kifst.hr; 3 Clinical Department of Diagnostic and Interventional Radiology, University Hospital of Split, 21000 Split, Croatia; goran.dujic@mefst.hr; 4 Department of Integrative Physiology, School of Medicine, University of Split, 21000 Split, Croatia; zeljko.dujic@mefst.hr; 5 High Performance Sport Center, Croatian Olympic Committee, 10000 Zagreb, Croatia

**Keywords:** DAST, SAST, RAST, specific anaerobic capacities, freedivers

## Abstract

Breath-hold diving is explained as an activity that requires enduring muscle asphyxia and acidosis, high anaerobic capacity, and the tactic of the dive. Therefore, this study aimed to construct and validate tests that will mimic anaerobic processes in the specific media of freedivers. The sample of participants included 34 Croatian freedivers (average age: 26.85 ± 4.0 years, competitive age: 3.82 ± 1.92 years, their body height: 180.14 ± 8.93 cm, and their body mass: 76.82 ± 12.41 kg). The sample of variables consists of anthropometric indices, competitive efficiency (maximal length of a dive (DYN)), and specific anaerobic capacities (100 m and 2 min tests). Newly developed tests included the swimming anaerobic sprint test (SAST) and diving anaerobic sprint test (DAST). DAST and SAST variables included the total time of the test (DAST/SAST) and the fastest interval (DAST_max_/SAST_max_). The results showed good reliability of the tests with high Cronbach alpha coefficients (DAST: 0.98, DAST_max_: 0.97, SAST: 0.99, SAST_max_: 0.91). Furthermore, pragmatic validity shows a high correlation among all variables and DAST (DYN: −0.70, 100 m: 0.66, 2 min: −0.68). High relation is also found between 100 m (0.96), 2 min (−0.94), and a moderate result for DYN (−0.43) and the SAST test. A factor analysis extracted one significant factor. The factor analysis involved DAST, SAST, DYN, 100 m, and 2 min tests regarding factor 1. After the examination of all variables, the total time of the DAST test showed the best predictive values for the performance of divers. However, both tests could be used for diagnostics and the evaluation of specific condition abilities in freediving.

## 1. Introduction

Freediving is an activity in which participants try to achieve maximal time, distance, or depth in a single breath [1]. Since freediving became more popular, many athletes have competed in international competitions. The governing bodies of all competitions in freediving are CMAS (Confédération Mondiale des Activités Subaquatiques) and AIDA (Association Internationale pour le Développement de l’Apnée). CMAS and AIDA divide diving disciplines into two main components: dynamic (maximal depth or length) and static (maximal time). Static apnea (STA) is the only discipline based on time, i.e., the longest duration a diver can hold their breath, while immersed. The greatest challenge during STA is to minimize oxygen consumption, despite extreme and cumulative hypercapnia and hypoxemia and an overwhelming urge to breathe. Dynamic apnea (DA) is the discipline whereby divers swim horizontally underwater for a maximal distance usually in a pool or in the open sea, with or without fins. These long periods without oxygen are known as apnea or breath-hold diving (BHD).

BHD has been previously defined as enduring apnea for more than 4 min. It is characterized by resistance of reactive oxygen species, reduced sensitivity to hypoxia, and low mitochondrial oxygen consumption [1]. Furthermore, it was hypothesized about humans’ diving ability, mainly because of the necessity for humans to perform apnea. Today, breath-hold-, apnea-, or freediving is practiced by recreational divers, seafood divers, military divers, and competitive athletes [2,3]. The mammalian dive response (DR) that is initiated during apnea or facial submersion in water includes peripheral vasoconstriction, a reduced cardiac output (CO), bradycardia, and low muscle oxygenation, whereas cerebral perfusion is augmented as hypercapnia develops [4,5,6,7,8]. Prolonged periods of apnea are the main reason for the extremity of freediving and therefore the potential danger that is set upon the athletes [9,10]. No matter the risks of apnea, freedivers are constantly trying to break records in all disciplines. Therefore, the proper preparation of all aspects and capacities of athletes is necessary.

Previous studies determined several important factors for success in freediving. Precisely, freediving demands the capacity of oxygen that is stored in the body (e.g., lungs, blood, tissues), tolerance of cerebral asphyxia, metabolism capacity, and level of the diving response activation [11]. Furthermore, while divers are moving through the water, the working economics and technique are also necessary. Following that, there is a high need for enduring muscle asphyxia and acidosis, high anaerobic capacity, and the tactic of the dive [12]. Even though different disciplines demand different performance factors, the accumulations of CO_2_, lactates, hypoxia, and muscle asphyxia are the main limiting factors of the athletes. These limitations may lead to a loss of muscle strength, which is important for the propulsion of the fins during dynamic dives. Therefore, it is proposed that divers develop repetitive strength and endurance of the lower limb muscles. These training regimes should increase tolerance of asphyxia and acidosis during the dive. Taking into account all of the above-mentioned factors, freediving is considered an anaerobic sport. This implication can be seen through the fact that high values of lactate levels have been examined previously [12,13], and anaerobic glycolysis was found as a main energy source during prolonged deep dives [14,15]. Therefore, the evaluation of these anaerobic capacities in freediving could serve as a good assessment tool for performance.

A literature review showed that there are no studies that developed tests specific for freedivers. Therefore, an analysis of sport-specific tests showed one test that is performed for the examination of anaerobic capacities on dry land. RAST (Running Anaerobic Sprint Test) is performed in a manner where the participants perform six sprints (35 m) with the maximal velocity with 10 s rests in between [16]. RAST was shown to be a valid assessment test for anaerobic metabolism. Therefore, this study aimed to construct and validate tests similar to RAST that will mimic anaerobic processes in the specific media of freedivers. Also, it should be highlighted that this study aimed at dynamic apnea.

## 2. Materials and Methods

### 2.1. Sample of Participants

The sample of participants included 34 elite and moderate (10 females) Croatian freedivers. The average age of participants was 26.85 ± 4.0 years, the average competitive age was 3.82 ± 1.92 years, their body height was 180.14 ± 8.93 cm, and their body mass was 76.82 ± 12.41 kg. The sample consisted of 6 elite freedivers (3 female) who were record holders and/or medalists at European and world championships in static and dynamic apnea disciplines. Other divers were moderate competitive divers that competed for at least 1 year. At the time of this study, all participants were healthy and did not have any injuries or illnesses. Before the beginning of the procedure, they were informed about all the risks and possible dangers of the procedure. They also signed written consent. All experimental procedures were completed following the Declaration of Helsinki 2008, and they were approved by the corresponding authors’ institutional research ethics board (Ethics Board Approval No. 2181-205-02-05-22-035).

### 2.2. Sample of Variables

The sample of variables consists of anthropometric indices, competitive efficiency, and specific anaerobic capacities.

Anthropometric indices were used solely for descriptive purposes of this study and they included body height and body mass.

Competitive efficiency was determined as a maximal result in the competitive dynamic diving discipline. The result presented was the maximal length of a dive performed in a swimming pool. The divers were equipped with a monofin during a dynamic apnea test and were instructed to give maximal effort in one dive.

Specific anaerobic capacities included measurement of 100 m crawl style maximal swimming (100 m), 2 min of maximal crawl swimming (2 min), and 2 newly developed tests: swimming anaerobic sprint test (SAST) and diving anaerobic sprint test (DAST). Swimming tests (100 m and 2 min) were performed without the jump start. The test started after the start signal was given. The 100 m test measures the fastest time to swim 100 m, and the 2 min test measures the length that the diver achieved after 2 min of swimming. Tests DAST and SAST were used to measure specific anaerobic capacities in the water. They were developed following the RAST (Running Anaerobic Sprint Test), which was developed for the anaerobic capacity of runners [16].

### 2.3. Procedure

The participants took the test in a closed 25 m long swimming pool. After arrival, the participants were acquainted with the tests and undertook a standardized whole-body warm-up and an individual apnea warm-up. The test–retest routine was performed with a 7-day break in between, for DAST and SAST. Also, the tests were performed on different days so that participants had an appropriate break.

The procedure of DAST was performed in a manner where divers were facing toward the direction of a dive with one arm holding the edge of the pool. The participant was equipped with a swimming suit, mask, and a monofin. After 5 s, the diver was signaled to start with 6 × 25 m dives with the maximal speed with a 15 s rest in between dives. The time of every interval was measured. The result of the test was calculated as a collected time during the test, without taking into account the rest between dives. Also, the fastest interval is derived from the results.

The SAST test was performed in the same manner as the DAST, except the divers needed to use the swim crawl technique during the intervals. Also, SAST was performed with a swimming technique (without fins), and the participants were allowed to breath normally.

### 2.4. Statistics

The non-parametric/parametric nature of the variables was tested using the Shapiro–Wilk’s test procedure. The calculation of the descriptive statistic parameters included means and standard deviations. The reliability and pragmatic validity were defined with the Pearson R correlation coefficient. A factor analysis was shown to define the variability of the tests and their connection with other anaerobic tests. Furthermore, the Bland–Altmann approach determined the differences and proportional bias between measurements. For the determination of prediction in the DYN variable, multiple linear regression and the Durbin–Watson method were used. For the calculation of homoscedasticity, the Breusch–Pagan test was performed.

Statistica ver. 13.0 (Dell Inc., Austin, TX, USA) and Statistical Package for Social Science (SPSS), ver. 27 (SPSS, Chicago, IL, USA) were used for the analyses, and a level of 95% (*p* < 0.05) was applied.

## 3. Results

Table 1 shows the results of descriptive statistics for DAST, SAST, DYN, 100 m, and 2 min tests. Presented results are means with standard deviations, and minimal and maximal results. Also, for DAST and SAST tests, the Cronbach alpha is presented as a value of reliability. Precisely, the Cronbach alpha coefficient is very high for all four variables (DAST: 0.98, DAST_max_: 0.97, SAST: 0.99, SAST_max_: 0.91).

**Figure 1:**
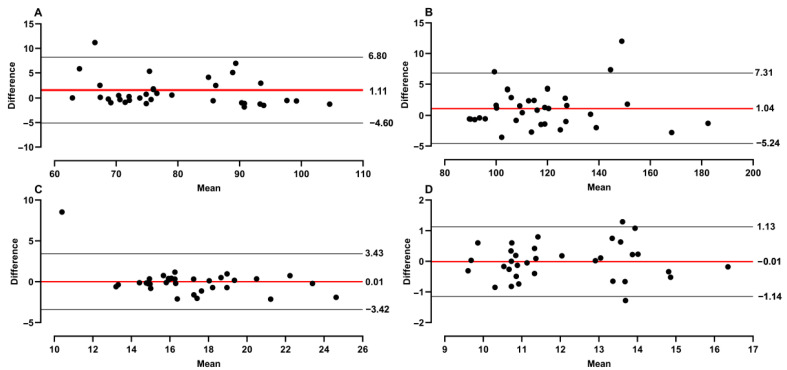
Bland–Altman plot between means and differences of measured tests, with bias (red line) and upper and lower limits (black lines); (**A**) DAST, (**B**) SAST, (**C**) DAST_max_, (**D**) SAST_max_.

Figure 1 presents a Bland–Altman plot with lower and upper bounds, and bias of differences between measurements in all variables of tests (DAST and SAST). The DAST test showed a proportional bias of 1.11, whereas the SAST bias was 1.04. A bias of 0.01 is presented in both DAST_max_ and SAST_max_. Furthermore, it can be noted that in both DAST variables, only one participant is out of bounds in the plot. On the other hand, the plot for SAST defines two participants above the upper or lower bound.

Correlations between DAST and SAST measurements: It can be seen that all correlations show a significant correlation from a 0.46 to 0.97 coefficient (see Table 2). Furthermore, the factor analysis extracted one significant factor. The factor involved both DAST and SAST measurements with a relation ranging from −0.85 to −0.94. Also, DYN (0.64), 100 m (−0.92), and 2 min (0.92) tests were batched in factor 1. The explained variance of the factor is 8.13, with a proportion total of 0.74 (Table 3).

Table 4 demonstrates correlations between DAST and SAST tests with specific anaerobic tests (100 m and 2 min) and competitive efficiency in breath-holding diving (DYN). The correlations show a high relation among all variables and DAST (DYN: −0.70, 100 m: 0.66, 2 min: −0.68). Furthermore, a high relation is also found for 100 m (0.96) and 2 min (−0.94), and a moderate relation for DYN (−0.43), with the SAST test. Similar results can be observed for the fastest interval of the divers for both DAST (DYN: −0.70, 100 m: 0.66, 2 min: −0.68) and SAST (DYN: −0.34, 100 m: 0.91, 2 min: −0.89).

The analysis of Table 5 demonstrates a small Durbin–Watson coefficient of 1.35 and a high regression coefficient of 0.74. Furthermore, only the DAST variable showed significance in the model (*p* = 0.02), with a B coefficient of −4.31 and a β coefficient of −1.22. Table 6 demonstrates the homoscedasticity of the means in the variables of DAST and SAST (*p* = 0.26).

## 4. Discussion

Capacities of specific conditions play a significant role in every athlete’s training regime and performance. The diagnostics of such capacities is a valuable and important tool for evaluating the athlete. Following that, the diagnostic process is maintained for the appropriate testing instruments. A literature review showed that such tools are non-existent in the evaluation of anaerobic endurance for breath-hold athletes. Also, there seems to be a high need for the determination of technical and motorical adaptation to water (aquacity), which is important for dynamic discipline in freediving. Therefore, this study tried to evaluate and determine the metric characteristics of two newly developed and constructed anaerobic tests: DAST (diving anaerobic sprint test) and SAST (swimming anaerobic sprint test).

The analysis of the results between the test and retest measurement of DAST showed a high correlation for tested variables of total time and fastest interval. Additionally, variable DAST_max_ presented a high correlation (0.95) and α (0.97). Similar results are found for the variable of total time for DAST (r = 0.97; α = 0.98). These results present the high reliability of this newly developed test for freedivers. Precisely, it took divers to dive a distance of 25 m between 9 and 16 s, which shows the anaerobic nature of this variable. Also, in terms of energy used for such a process, the duration implies a usage of phosphate reserves. Furthermore, the full DAST test represents a total time to dive six intervals of 25 m, and the results are from 61 to 105 s. Therefore, this variable dominantly measures the glycolytic component of specific anaerobic capacities of freedivers. Since a monofin was used to finish this test, it could be connected to the results of dynamic competitive discipline. This is mainly because this discipline lasts between 73 and 235 s. Previously, it was established that the metabolic response during the dive is mainly anaerobic [11,17,18,19,20,21,22]. Even though the competitive dives last longer than the DAST test, it still can be concluded that glycolytic metabolism will be prolonged during the dive. With that in mind, this test could be used to evaluate specific anaerobic ability for dynamic divers.

Additionally, repeated measurements of the SAST test showed somewhat similar results to DAST. A statistically significant correlation was found between the SAST total time variable (0.99) and the fastest interval of SAST (0.83). These findings implicate the high reliability of new anaerobic tests for swimming performance. Same as was presented for DAST, the full SAST test lasts from 89 to 183 s and its fastest interval is from 13 to 26 s. These times implicate the usage of glycolytic metabolism, for the full test, and phosphate metabolism, for one interval of the test. Even though crawl swimming is not a part of diving, such tests could be used for training and the development of anaerobic capacities [23].

Apart from the reliability, pragmatic validity results present a high correlation between both DAST and SAST tests and competitive efficiency tests (DYN, 100 m, and 2 min). Precisely, DAST correlated significantly with all three tests (first measurement, −0.70; 0.66; −0.68; second measurement, −0.72; 0.62; −0.63). These results imply that this test is connected with all variables that represent the swimming and diving capacities of the participants. Therefore, DAST is a valid test to determine the specific performance of the divers. Similar results are presented for the fastest interval of DAST, which is logical due to the fact that it shows anaerobic capacity. For the SAST test, there is a visibly smaller relation with the DYN. This result is present due to the fact that SAST is performed with swimming movements that are substantially different than movements an athlete performs during the dives. Therefore, lack of correlation with diving capacities is not unexpectable. On the other hand, there is a high connection of all SAST variables from both measurements with 100 m and 2 min tests. Similarly, the multiple linear regression model showed a high coefficient of tests in the prediction of DYN. Precisely, when DAST, SAST, DAST_max_, and SAST_max_ parameters are placed in the prediction model, only DAST showed significant correlation with DYN. Such observation corresponds with previous conclusions, since the SAST test is swimming-dependent. Following all that is mentioned above, it can be concluded that DAST is a valuable tool for dynamic apnea prediction.

However, both DAST and SAST could be used as reliable and valid tools for the assessment of divers’ anaerobic capacities. Such conclusions can be observed through the factor analysis, which extracted one factor containing all variables of DAST and SAST, together with DYN, 100 m, and 2 min tests. Together with a high correlation of variables with this factor, there is a high explained variance (8.13) and total proportion (0.74). Accordingly, this factor presents aquatic anaerobic ability of the participants. It also confirms factorial validity of newly constructed tests.

## 5. Conclusions

Following the aim of this study, it presented a high validity and reliability of these newly developed tests for the determination of specific anaerobic freediving performance. Also, high correlations between the results of the tests and competitive efficiency variables show a significant role of anaerobic metabolism in the determination of success in dynamic disciplines of freediving. After the examination of all variables extracted from the DAST and SAST, the total time of the DAST test showed the best predictive values for the performance of divers. However, both tests could be used for diagnostics and the evaluation of specific condition abilities in freediving. Additionally, tests could be used for the prediction of performance and as a selection criterion for different disciplines of freediving. On the other hand, the results of this study are restricted to the characteristics of the studied population and modality (dynamic apnea).

## Figures and Tables

**Table 1:** Descriptive statistics and reliability coefficient between first and second measurements of DAST and SAST, dynamic apnea, 100 m sprint, and 2 min swimming test.

Variable	Mean	SD	Min	Max	α
DAST_1_ (s)	80.49	11.14	62.92	103.97	0.98
DAST_2_ (s)	79.38	11.76	60.95	105.20	
DAST_1max_ (s)	12.08	1.77	9.45	16.26	0.97
DAST_2max_ (s)	12.09	1.74	9.56	16.44	
SAST_1_ (s)	119.05	22.07	89.34	181.82	0.99
SAST_2_ (s)	118.02	21.78	89.91	183.10	
SAST_1max_ (s)	17.07	2.68	12.90	23.67	0.91
SAST_2max_ (s)	17.06	6.13	25.58	3.41	
DYN (m)	113.05	40.29	50.00	200.00	-
100 m (s)	83.85	16.56	61.00	130.00	-
2 min (m)	136.35	20.93	92.00	175.00	-

SD, standard deviation; Min, minimal result; Max, maximal result; α, Cronbach alpha coefficient; DAST_1_, diving anaerobic sprint test 1st measurement; DAST_2_, diving anaerobic sprint test 2nd measurement; SAST_1_, swimming anaerobic sprint test 1st measurement; SAST_2_, swimming anaerobic sprint test 2nd measurement; DAST_1max_, diving anaerobic sprint test fastest interval of 1st measurement; DAST_2max_, diving anaerobic sprint test fastest interval of 2nd measurement; SAST_1max_, swimming anaerobic sprint test fastest interval of 1st measurement; SAST_2max_, swimming anaerobic sprint test fastest interval of 2nd measurement.

**Table 2:** Correlations between first and second measurements of DAST and SAST.

Variable	DAST_2_ (s)	DAST_2max_ (s)	SAST2 (s)	SAST_2max_ (s)
DAST_1_ (s)	0.97	0.93	0.70	0.53
DAST_1max_ (s)	0.95	0.95	0.70	0.52
SAST_1_ (s)	0.65	0.64	0.99	0.86
SAST_1max_ (s)	0.46	0.45	0.92	0.83

DAST_1_, diving anaerobic sprint test 1st measurement; DAST_2_, d 2nd measurement; SAST_1_, swimming anaerobic sprint test 1st measurement; SAST_2_, 2nd measurement; DAST_1max_, diving anaerobic sprint test fastest interval of 1st measurement; DAST_2max_, 2nd measurement; SAST_1max_, swimming anaerobic sprint test fastest interval of 1st measurement; SAST_2max_, 2nd measurement.

**Table 3:** Factor analysis for first and second measurements of DAST and SAST, dynamic apnea, 100 m sprint, and 2 min swimming test.

Variable	Factor 1
DAST_1_ (s)	−0.88
DAST_1max_ (s)	−0.86
DAST_2_ (s)	−0.85
DAST_2max_ (s)	−0.83
SAST_1_ (s)	−0.94
SAST_1max_ (s)	−0.83
SAST_2_ (s)	−0.94
SAST_2max_ (s)	−0.80
DYN (m)	0.64
100 m (s)	s0.92
2 min (m)	0.92
Explained variance	8.13
Proportion total	0.74

DAST_1_, diving anaerobic sprint test 1st measurement; DAST_2_, diving anaerobic sprint test 2nd measurement; SAST_1_, swimming anaerobic sprint test 1st measurement; SAST_2_, swimming anaerobic sprint test 2nd measurement; DAST_1max_, diving anaerobic sprint test fastest interval of 1st measurement; DAST_2max_, diving anaerobic sprint test fastest interval of 2nd measurement; SAST_1max_, swimming anaerobic sprint test fastest interval of 1st measurement; SAST_2max_, swimming anaerobic sprint test fastest interval of 2nd measurement; DYN, maximal dynamic apnea; 100 m, 100 m swimming sprint; 2 min, 2 min maximal swimming.

**Table 4:** The correlations between first and second measurements of DAST and SAST, dynamic apnea, 100 m sprint, and 2 min swimming tests.

Variable	DYN (m)	100 m (s)	2 min (m)
DAST_1_ (s)	−0.70	0.66	−0.68
DAST_1max_ (s)	−0.63	0.66	−0.64
DAST_2_ (s)	−0.72	0.62	−0.63
DAST_2max_ (s)	−0.67	0.62	−0.60
SAST_1_ (s)	−0.43	0.96	−0.94
SAST_1max_ (s)	−0.34	0.91	−0.89
SAST_2_ (s)	−0.44	0.95	−0.93
SAST_2max_ (s)	−0.34	0.83	−0.85

DAST_1_, diving anaerobic sprint test 1st measurement; DAST_2_, diving anaerobic sprint test 2nd measurement; SAST_1_, swimming anaerobic sprint test 1st measurement; SAST_2_, swimming anaerobic sprint test 2nd measurement; DAST_1max_, diving anaerobic sprint test fastest interval of 1st measurement; DAST_2max_, diving anaerobic sprint test fastest interval of 2nd measurement; SAST_1max_, swimming anaerobic sprint test fastest interval of 1st measurement; SAST_2max_, swimming anaerobic sprint test fastest interval of 2nd measurement; DYN, maximal dynamic apnea; 100 m, 100 m swimming sprint; 2 min, 2 min maximal swimming.

**Table 5:** Linear regression model between DYN variable and tests (DAST and SAST).

Model	R	R^2^	Adjusted R^2^	Std. Error of the Estimate	Durbin–Watson
	0.74	0.54	0.48	29.09	1.35
Variable	B	Std. Error	β	T	*p*
DAST (s)	−4.31	1.73	−1.22	−2.49	0.02 *
SAST (s)	0.69	0.74	0.38	0.94	0.35
DAST_max_ (s)	9.13	11.36	0.39	0.80	0.43
SAST_max_ (s)	−3.72	4.61	−0.27	−0.81	0.43

R, linear regression coefficient; R^2^, linear regression coefficient squared; B, unstandardized coefficient of correlation; β, standardized coefficient of correlation; T, test value; *p*, level of significance; *, significance set at *p* < 0.05; DAST, diving anaerobic sprint test; SAST, swimming anaerobic sprint test; DAST_max_, diving anaerobic sprint test fastest interval; SAST_max_, swimming anaerobic sprint test fastest interval.

**Table 6:** Homoscedasticity analysis of DAST and SAST tests.

Model	Sum of Squares	Mean Square	F	*p*
Regression	5,455,232.29	1,363,808.07	1.28	0.26
Residual	30,951,997.08	1,067,310.24		
Total	36,407,229.37			

F, test value; *p*, level of significance.

## Data Availability

Data are contained within the article.
